# Prehospital characteristics among patients with sepsis: a comparison between patients with or without adverse outcome

**DOI:** 10.1186/s12873-019-0255-0

**Published:** 2019-08-06

**Authors:** Agnes Olander, Henrik Andersson, Annelie J. Sundler, Anders Bremer, Lars Ljungström, Magnus Andersson Hagiwara

**Affiliations:** 1 PreHospen – Centre for Prehospital Research, University of Borås, Allégatan 1, SE- 405 30 Borås, Sweden; 2 Faculty of Caring Science, Work Life and Social Welfare, University of Borås, Borås, Sweden; 3 Faculty of Health and Life Sciences, Linnaeus University, Växjö, Sweden; 4 Department of Infectious Diseases, Institute of Biomedicine, Sahlgrenska Academy, University of Gothenburg, Gothenburg, Sweden; 5 Department of Infectious Diseases, Skaraborg Hospital, RegionVästra Götaland, Skövde, Sweden

**Keywords:** Emergency medical services, Characteristics, Prehospital, Sepsis, Symptoms, Vital signs

## Abstract

**Background:**

The prehospital care of patients with sepsis are commonly performed by the emergency medical services. These patients may be critically ill and have high in-hospital mortality rates. Unfortunately, few patients with sepsis are identified by the emergency medical services, which can lead to delayed treatment and a worse prognosis. Therefore, early identification of patients with sepsis is important, and more information about the prehospital characteristics that can be used to identify these patients is needed. Based on this lack of information, the objectives of this study were to investigate the prehospital characteristics that are identified while patients with sepsis are being transported to the hospital by the emergency medical services, and to compare these values to those of the patients with and without adverse outcomes during their hospital stays.

**Methods:**

This was a retrospective observational study. The patients’ electronic health records were reviewed and selected consecutively based on the following: retrospectively diagnosed with sepsis and transported to an emergency department by the emergency medical services. Data were collected on demographics, prehospital characteristics and adverse outcomes, defined as the in-hospital mortality or treatment in the intensive care unit, and analysed by independent sample t-test and chi-square. Sensitivity, specificity and likelihood ratio, of prehospital characteristics for predicting or development of adverse outcome were analysed.

**Results:**

In total, 327 patients were included. Of these, 50 patients had adverse outcomes. When comparing patients with or without an adverse outcome, decreased oxygen saturation and body temperature, increased serum glucose level and altered mental status during prehospital care were found to be associated with an adverse outcome.

**Conclusions:**

The findings suggests that patients having a decreased oxygen saturation and body temperature, increased serum glucose level and altered mental status during prehospital care are at risk of a poorer patient prognosis and adverse outcome. Recognizing these prehospital characteristics may help to identify patients with sepsis early and improve their long-term outcomes. However further research is required to predict limit values of saturation and serum glucose and to validate the use of prehospital characteristics for adverse outcome in patients with sepsis.

**Electronic supplementary material:**

The online version of this article (10.1186/s12873-019-0255-0) contains supplementary material, which is available to authorized users.

## Background

The early identification of patients with sepsis in the prehospital setting is crucial for reducing the adverse outcome risks due to inadequate assessments or delayed medical interventions [1]. Therefore, it is necessary for clinicians in the emergency medical services (EMS) to correctly assess and interpret the prehospital characteristics of these patients.

Sepsis is a common and serious worldwide medical condition [2–4]. The incidence in Sweden of severe sepsis according to Sepsis-2 criteria is 687/100,000 inhabitants/year, and the incidence of the newly developed definition Sepsis-3 is 780/100,000 inhabitants/year [4]. Approximately 50 to 75% of the patients with sepsis are cared for and transported to emergency departments (EDs) by the EMS [5–7]. Unfortunately, these patients have a high in-hospital mortality rate [1, 5–7] and relatively few patients (only 6 to 36%) are suspected of having sepsis by EMS clinicians in the prehospital field [1, 6, 8, 9]. However, the clinical presentation of sepsis is often nonspecific [10], making early identification difficult [11], which may lead to delayed treatment and a worse prognosis.

The definition of sepsis recently changed. Previously, sepsis (Sepsis-2) was defined as a suspected infection in combination with the systemic inflammatory response syndrome (SIRS) [12, 13]. Sepsis with organ dysfunction was termed severe sepsis or septic shock if also hypotensive and not responding to fluid challenge [12, 13]. Since 2016 sepsis is defined as ‘life-threatening organ dysfunction caused by a dysregulated host response to infection’ commonly known as Sepsis-3 [14].

Early identification of sepsis by the EMS clinicians is crucial for these patients [15, 16]. Previous studies have indicated that a documented suspicion of sepsis in the EMS electronic health records shortened the time until the administration of antibiotics [1, 6] and a delayed time to the start of antibiotics is associated with increased progression of severe sepsis to septic shock and increased mortality [17]. These results indicate that a documented suspicion by the EMS are important for time to treatment in patients with sepsis. Prehospital screening tools for the identification of patients with sepsis and/or severe sepsis include the PREhospital Severe Sepsis (PRESS) score, Robson screening tool, Sepsis Alert protocol, quick Sequential (Sepsis-related) Organ Failure Assessment (qSOFA) and BAS 90–30-90 (based on the oxygen saturation, respiratory rate and systolic blood pressure) [8, 14, 18–20]. Despite the prehospital screening tools and treatment guidelines that are available, there is still extensive patient morbidity and mortality due to sepsis [14]. A previous study indicate that hospital treated sepsis is the leading cause of mortality worldwide [3] and patients with sepsis arriving to the ED trough EMS were more likely to have severe grades of sepsis, and more often admitted to the ICU than those arriving by other means [5, 7].

Symptoms and vital signs tend to vary among patients with sepsis. For example, recent Swedish studies have indicated that symptoms as respiratory difficulty, an altered mental status, nausea, diarrhoea and/or vomiting, severe localized pain, muscle weakness, a lack of energy, fever and/or chills were common among patients with severe sepsis [21, 22]. However, in one previous study, only a small percentage of the patients with severe sepsis and septic shock were hypotensive according to the EMS records (14% with a systolic blood pressure of < 90 mmHg and 10% with a mean arterial pressure of *<* 65 mmHg) [23]. Another confounding vital sign is the body temperature, which does not rise in all patients with sepsis, making the diagnosis even more difficult [24]. Normal vital signs tend to be associated with a lower level of monitoring while the patient is being cared for in the ED, which may increase the risk of unnoticed deterioration and nonspecific symptoms tend to be associated with less favourable outcomes [25]. Vital signs and symptoms tend not to be specific to identify patients with sepsis and further research is required in the field.

Today, there is limited knowledge regarding the prehospital characteristics of patients with sepsis. To our knowledge, there is no previous study that has compared the prehospital characteristics of patients with sepsis with and without adverse outcomes during the hospital stay. Further research into the differences in the characteristics observed during the prehospital phase of sepsis is required, particularly in those patients at risk for poorer prognoses. Therefore, the objectives of the present study were to investigate the prehospital characteristics of patients with sepsis while they were being transported by the EMS, and to compare these values to those of the patients with and without adverse outcomes during their hospital stays.

## Methods

### Study design

A retrospective observational design was used for this study. Data was collected in 2017 from EMS and hospital electronic health records of the patients who retrospectively fulfilled the sepsis-2 diagnosis.

### Population and study setting

The data was collected from an epidemiological study database of patients with sepsis [26]. In this database, the patients has been retrospectively diagnosed with sepsis based on a modified version of Sepsis-2 criteria [10, 27] (see Additional file 1: Table S1 for details). All data for the present study was collected from this database by the first author.

A convenience sample of patients were included from those patients admitted to the hospital during a four-month period (1 January until 30 April 2012). These months were chosen because there tends to be a higher frequency of infections at this time of the year [28]. The inclusion criteria were: adult patients ≥18 years old, admitted to the Skaraborg Hospital in western Sweden, who within 48 h received intravenous antibiotic treatment on suspicion of sepsis, transported by the EMS and retrospectively diagnosed with sepsis. A total of 327 patients met the inclusion criteria.

Skaraborg Hospital is a 640-bed secondary care hospital serving a population of 256,700 inhabitants. The EMS organization is comprised of nine ambulance stations performing 35,000 hospital admissions annually. According to the national requirements, each ambulance unit consists of healthcare professionals who are trained in advanced life support and is authorized to prepare and administer pharmaceuticals [29].

### Data collection

The data protocol was constructed using Microsoft Access 2016 (Microsoft, Redmond, Washington, USA) in order to read and extract the data from the electronic health records of the EMS and hospital in a systematic and objective manner. Based on this data protocol, the data was analysed using IBM SPSS Statistics for Windows (version 21; IBM Corp., Armonk, New York, USA). When applying the data protocol to the first 18 electronic health records, five researchers evaluated the usability of the protocol in order to ensure its validity, enhance the objectivity and avoid the need for subjective interpretations. During this evaluation, some vague terminology and expressions were found regarding the prehospital characteristics. These were discussed and compared to the academic literature in order to determine more explicit definitions, thus enabling objective interpretations. After this, retrospective collection of data was conducted by the first author.

The data collection protocol was divided into four segments. The first part consisted of a creating a code for tracing the data back to the relevant electronic health records. The second part covered the demographic characteristics, including the gender, age, marital status, form of housing, type of home assistance and previous medical history. The third part covered the patients’ prehospital characteristics while they were being transported by the EMS, such as the vital signs, laboratory test results and symptoms. The fourth part covered the patient outcomes during the hospital stay.

### Statistical analysis

For the baseline data, all of the continuous variables were expressed as the mean and standard deviation (SD), while the categorical data was expressed as the number (n) and percentage (%). In order to compare the patients with and without adverse outcomes, they were divided into two groups. Those patients who were cared for in the intensive care unit (ICU) or who died during the hospital stay (as a result of sepsis) formed the adverse outcome patient group. The other patients formed the group of patients without adverse outcomes. In order to determine the differences between the groups, an independent t-test was used for the continuous data and a chi-squared test was used for the categorical data. The continuous data were approximately normally distributed. A *p*-value of < 0.05 was considered to be statistically significant, and those variables with *p*-values of < 0.05 were also included and analysed in a final model including sensitivity, specificity, and likelihood ratio, both positive and negative. To carry out these analyses, the variables saturation, temperature and serum glucose were dichotomized. Limit values for saturation < 90% and temperature > 38C° were selected based on SIRS criteria [12, 13]. The limit value for serum glucose > 11 mmol/l was selected based on previous studies showing that hyperglycemia > 11 mmol/l may affect the outcome of patients with sepsis at ICU [30]. For all of the statistical analyses and data processing, IBM SPSS Statistics for Windows was used.

## Results

### Demographic

A total of 327 patients were included in this study. The mean age of the patients was 75 years old and 175 (54%) were males, for demographic see Table 1.

**Table 1:** Chi-squared test on patient’s demographics

Demographics	All patients (*n* = 327) Mean (SD)/n (%)	Patients without adverse outcome (*n* = 277) Mean (SD)/n (%)	Patient with adverse outcome (*n* = 50) Mean (SD)/n (%)	*P-*value
Age (y)	75 (15)	75 (16)	74 (12)	0.74
Gender				
Male	175 (54%)	145 (52%)	30 (60%)	0.32
Female	152 (46%)	132 (48%)	20 (40%)	0.32
Co-morbidity				
Coronary artery disease/ congestive heart failure	139 (43%)	112 (40%)	27 (54%)	0.07
Hypertension	102 (31%)	88 (32%)	14 (28%)	0.60
History of infections^a^	83 (25%)	72 (26%)	11 (22%)	0.55
Diabetes mellitus	73 (22%)	58 (21%)	15 (30%)	0.16
VChronic obstructive pulmonary disease	56 (17%)	44 (16%)	12 (24%)	0.16
Malignancy	49 (15%)	45 (16%)	4 (8%)	0.13
Cerebrovascular disease/ stroke	48 (15%)	42 (15%)	7 (14%)	0.83
Immunosuppress	32 (10%)	29 (11%)	3 (6%)	0.33
No co-morbidity	23 (7%)	21 (8%)	2 (4%)	0.36
Asthma	14 (4%)	14 (5%)	0 (0%)	0.10
Epilepsy	5 (2%)	4 (1%)	1 (2%)	0.77

^a^History of an infection within last month

Among the patients, 277 (85%) were without adverse outcomes, while 50 (15%) of the patients exhibited adverse outcomes. Out of the 277 patients without adverse outcome 235 (85%) hade sepsis and 42 (15%) severe sepsis. Out of the patients with adverse outcome, 29 (58%) were treated in the ICU and 21 (42%) died during the hospital stay. Of the patients with adverse outcome, 4 (8%) patients had sepsis 36 (72%) severe sepsis and 10 (20%) septic shock. There were no significant differences between the patients without adverse outcome and those with adverse outcome regard to age, gender or comorbidities, see Table 1.

An early suspicion of sepsis, as documented by the EMS clinicians, was found in 36 (11%) out of the 327 patients with sepsis. When comparing patients without and with adverse outcome, the EMS clinicians suspected sepsis in 28 (10%) of the patients without adverse outcomes and 9 (18%) of the patients with adverse outcomes (*p* = 0.01).

### Prehospital characteristic

In all patients with sepsis, the symptoms most commonly documented in the EMS health records were sudden respiratory difficulty in 219 (67%) and muscle weakness in 84 (26%). There were a significant difference in relation to the symptoms altered mental status and shivering when comparing the patients without and with adverse outcomes. Patients without adverse outcomes were more likely to have symptoms such as shivering during their prehospital care and patients with adverse sepsis outcome were more likely to have an altered mental status during their prehospital care, see Table 2.

**Table 2:** Chi-squared test on patient’s symptoms in the EMS

Symptoms	All patients *n* = 327 n (%)	Patients without adverse outcome *n* = 277 n (%)	Patient with adverse outcome *n* = 50 n (%)	*P-*value
Respiratory difficulties	219 (67%)	180 (65%)	39 (78%)	0.07
Muscle weakness^a^	84 (26%)	70 (25%)	14 (28%)	0.68
Gastrointestinal symptoms^b^	60 (18%)	53 (19%)	7 (14%)	0.39
Altered mental status^c^	58 (18%)	43 (15%)	15 (30%)	0.01^*^
Pain unspecified^d^	56 (17%)	45 (16%)	11 (22%)	0.32
Shivering	52 (16%)	49 (18%)	3 (6%)	0.04^*^
Chest discomfort	28 (9%)	26 (9%)	2 (4%)	0.21
Abdominal pain	28 (9%)	23 (8%)	5 (10%)	0.69

^a^Being unable to perform tasks requiring muscle strength that are usually done with ease in the patient’s daily life, e.g. unable to stand, falling and collapsing, fainting, lying on the floor

^b^Diarrhoea, vomiting or/and nausea

^c^Decreased consciousness, difficult to reach, drowsiness, not responding when spoken to, or answering inappropriately

^d^Pain from various areas in body and “pain out of proportion”

^*^ Significant (*p* = < 0.05)

For the distribution of vital signs and laboratory test among all patients, and between patients without or with adverse outcome, see Table 3. When comparing the vital signs and laboratory test results between the two groups, those patients with adverse outcomes had significant lower oxygen saturations, lower body temperatures and higher serum glucose levels, see Table 3.

**Table 3:** Independent t-test on vital signs and laboratory test in the EMS

Vital signs and laboratory test	All patients (*n* = 327) Mean/(SD)/n	Patients without adverse outcome (*n* = 277) Mean/(SD)/n	Patient with adverse outcome *n* = 50 Mean/(SD)/n	*P-*value
Min oxygen saturation (%)	89 (7) *n* = 323	90 (6) *n* = 273	84 (10) n = 50	< 0.01^*^
Max heart rate (beats/min)	103 (21) *n* = 319	103 (20) *n* = 272	106 (22) *n* = 47	0.36
Min SBP^a^ (mmHg)	140 (28) *n* = 314	141 (29) *n* = 267	133 (37) n = 47	0.10
Max RR^b^ (breaths/min)	27 (8) *n* = 309	27 (8) *n* = 261	29 (9) *n* = 48	0.05
Max body temperature (C°)	38.1 (1.1) *n* = 303	38.2 (1.1) *n* = 298	37.4 (1.3) n = 47	< 0.01^*^
Max serum glucose (mmol/l)	9.5 (3.8) *n* = 182	9.2 (3.1) *n* = 154	11.4 (6.3) *n* = 28	< 0.01^*^

^a^Systolic blood pressure

^b^Respiratory rate

^*^ Significant (*p* = < 0.05)

Sensitivity, specificity, positive likelihood ratio and negative likelihood ratio of altered mental status, shivering, saturation, temperature and serum glucose for predicting adverse outcome in patients with sepsis are outlined in Table 4.

**Table 4:** Sensitivity, specificity, positive likelihood ratio, negative likelihood ratio of prehospital characteristics for predicting adverse outcome in patients with sepsis

	Sensitivity, % (95% CI)	Specificity, % (95% CI)	Positive Likelihood Ratio, (95% CI)	Negative Likelihood Ratio, (95% CI)
Symptoms in EMS				
Altered mental status	35 (18–54)	84 (79–88.)	2.2 (1.2–3.8)	0.8 (0.6–1.0)
Shivering	7 (1–23)	83 (78–87)	0.4 (0.1–1.6)	1.1 (1.0–1.2)
Vital signs and laboratory test in EMS				
Min oxygen saturation < 90%	59 (39–77)	60 (54–65)	1.4 (1.0–2.0)	0.7 (0.4–1.1)
Max body temperature > 38C°	59 (39–78)	57 (51–63)	1.4 (0.9–1.9)	0.7 (0.4–1.1)
VMax serum glucose > 11 mmol/l	37 (16–62)	77 (70–83)	1.6 (0.8–3.1)	0.8 (0.6–1.1)

## Discussion

In the present study, the sepsis identification rate of the EMS clinicians, as documented in the patients’ electronic health records, was found to be low. This result corresponds with those of previous studies regarding the EMS clinician assessments of patients with sepsis and severe sepsis [1, 6, 8, 20, 31]. Additionally, our study also compared the EMS sepsis identification rate in relation to the patient outcomes. Unfortunately, challenges still remain with regard to the early recognition of patients with sepsis, without or with adverse outcomes. The prehospital identification of patients with sepsis can be difficult to establish [32]. The reason for these difficulties can only be speculated. One reason is limited medical knowledge of sepsis among EMS clinicians which can undermine the ability to correctly identify conditions in patients with sepsis [31, 33, 34]. Another reason is that EMS clinicians have suspected other conditions [32] . Finally, the difficulties could be related to the working conditions and the fact that EMS clinicians prioritize care of the patients rather than the documentation of observation and measurements carried out in the patient assessment [6, 7, 35]. However, research has shown that sepsis recognition training combined with the use of a screening tool could improve the ability of EMS clinicians to look for sepsis and identify these patients [19]. This underscores the value of education and clinical training in identifying relevant conditions when caring for patients with sepsis. It could also be an opportunity for EMS clinicians to increase the survival and decrease the morbidity of patients with sepsis in much the same manner as they do with other time-critical, life-threatening conditions, such as acute myocardial infarctions [36] and strokes [37].

EMS clinicians play important roles in the early assessment and identification of patients with sepsis, especially those patients at risk for adverse outcomes. In this study, oxygen saturation, serum glucose level, body temperature, shivering and mental status were found in the analyses to be abnormal in patients with sepsis. These prehospital characteristics may be important in the prehospital setting, where access to laboratory testing is limited. For example, lower oxygen saturations were related to adverse outcomes in patients with sepsis in this study. A lower oxygen saturation is known to be an important vital sign in patients developing severe sepsis [8, 11, 21], and it has been used as a comarker in several screening tools, such as BAS 90–30-90 and PRESS score [8, 11]. Still, when investigating whether a oxygen saturation < 90% could predict a development of adverse outcome, there was no clear likelihood of this. This may indicate that low oxygen saturation as a vital sign could identify patients with sepsis who are at risk for adverse outcomes, however which limit value that could predict adverse outcome requires further research. Another prehospital characteristic that was associated with an adverse outcome was an increased serum glucose level. However, when investigating whether a serum glucose > 11 mmol/l could predict a development of adverse outcome, there was no clear likelihood of this. Hyperglycaemia in critical illness cases, such as severe sepsis, has been shown to be a comarker of the illness severity and a predictor of a poor outcome in the ICU [30, 38–40]. A higher serum glucose level may be the result of the metabolic changes in the body caused by sepsis [41]. There have been no previous studies indicating that the serum glucose could be used during the prehospital assessment in patients suspected of having sepsis; therefore, further research is required in this area. The decrease in the oxygen saturation nor the increase in the serum glucose level seemed to be a result of a patient’s previous medical condition or comorbidity. When comparing the groups, there were no significant differences between them with regard to the prevalences of chronic obstructive pulmonary disease and diabetes mellitus, see Table 1.

An elevated body temperature has been associated with a lower in-hospital mortality rate in patients with severe sepsis. As the body temperature rises, so does the suspicion of sepsis, contributing to a faster recognition and treatment of this condition [42]. In the current study, those patients with adverse outcomes were found to have lower body temperatures when compared to those without adverse outcomes. Neither shows a temperature > 38 °C positive likelihood for predicting adverse outcome. A previous study indicating that many critically ill patients with sepsis do not have elevated body temperatures, [24] and hypothermia has been associated with sepsis-related in-hospital mortality [22]. However, further research is required on the body temperature in patients with sepsis and its impact on patients’ outcome.

In the present study patients with adverse outcome were found to have lower prevalence of shivering compared to those without adverse outcome and showed also a lower likelihood for predicting shivering in patients with adverse outcome. A previous study showed that a lower prevalence of shivering during the EMS period indicated a higher sepsis-related in-hospital mortality rate [22].

In the present study, an altered mental status was the only recognizable early symptom that was found to be associated with adverse patient outcomes in the analysis and indicated a positive likelihood to predict adverse outcome. These results are comparable to those of another study showing that an altered mental status during the EMS period indicated a higher sepsis-related in-hospital mortality rate [22]. Another study by Edman-Waller and colleagues [21] indicated that an altered mental status combined with a suspected infection should be taken as a warning sign that the patient may have or may be developing severe sepsis. Previous studies have advocated for acknowledging and integrating the symptom presentation into the sepsis identification screening tools [21, 22]. However, with the exception of an altered mental status, the results of the present study indicated that there may be some difficulty when using symptoms to identify patients with sepsis who are at risk for adverse outcomes.

## Limitations

This study was limited by the retrospective and convenience data collection from patients with sepsis in an epidemiological study database and symptoms and signs documented in patients electronic health records. This includes the inevitable uncertainty of assessing subjective symptoms based on the retrospective registration of data from these records. Another limitation was the small sample size of patients with adverse outcomes. This small sample size could lead to low statistical power and increases the risk that the null-hypothesis is rejected as false even though it is true.

Additionally, this study was conducted at a single centre, which could limit the generalizability of our findings. Another limitation was that data was collected from 2012. However, this was an explorative study in which the prehospital characteristics were described on the basis of a disease. Even though the presentation of sepsis may have been nonspecific [10], the specific characteristics have not changed over time. In several previous studies, the International Classification of Diseases (ICD) codes were used to define the diagnoses [20–22, 43]; however, this is known to consistently underestimate the prevalence [27]. By retrospectively determining whether the patients fulfilled the sepsis diagnosis in the electronic health records according to a suspect infection and ≥ 2 SIRS criteria, a larger number of patients with sepsis may have been included. If only the ICD codes were used to identify the sepsis patients, there may have been a higher percentage of severe sepsis patients and patients with symptoms more typical of the common picture of sepsis, because these patients are more readily identified in the clinical setting. Therefore, our methods may have increased the generalizability of the results of this study.

More prospective studies are needed to determine whether the prehospital characteristics that were identified are representative of patients with sepsis with adverse outcomes in the prehospital setting, and to understand their predictive value.

## Conclusions

The results of this study suggested that a low oxygen saturation and body temperature, high serum glucose level and altered mental status may be early prehospital characteristics that are related to poorer prognoses and adverse outcomes in patients with sepsis. The early recognition of prehospital characteristics by the EMS clinicians may affect the identification of these patients, and in the long run, the outcomes of the patients diagnosed with sepsis. However, it is not possible to predict limit values of saturation, body temperature and serum glucose to indicate adverse outcome in patients with sepsis. Further research is required to validate the use of these prehospital characteristics in the prehospital setting.

## Additional file

Additional file 1: **Table S1**. Sepsis criteria’s during study period. The supplementary file describes which sepsis criteria the patients were diagnosed with during the study period. (DOCX 15 kb)

## Data Availability

The datasets used and/or analysed during the current study are available from the corresponding author upon reasonable request.

## Abbreviations

ED: Emergency department
EMS: Emergency medical services
ICD: International Classification of Diseases
ICU: Intensive care unit
SIRS: Systemic Inflammatory Response Syndrome
