# Supplementary material for: Prehospital characteristics among patients with sepsis: a comparison between patients with or without adverse outcome
Source: BMC Emerg Med. 2019 Aug 6;19:43. doi: 10.1186/s12873-019-0255-0 (PMC6685242; doi:10.1186/s12873-019-0255-0)
Supplement: Supplementary file 1 — Table S1. Sepsis criteria’s during study period. The supplementary file describes which sepsis criteria the patients were diagnosed with during the study period. (DOCX 15 kb) [file 12873_2019_255_MOESM1_ESM.docx]

**Additional file 1: Table S1. Sepsis criteria’s during study period.**

| **Previous Swedish 2011 consensus definition based on Sepsis-2.**  The criteria presuppose that changes have occurred from a fairly normal organ function and are not expected to have other causes than the systemic inflammatory reaction. |
| --- |
| SIRS (systemic inflammatory response syndrome), ≥2 of   - Heart rate >90/min. - Respiratory rate >20/min. - Temperature >38.0^0^C or <36.0^0^C. - Leukocyte count >12.0 x 10^9^/ml or <4.0 x 10^9^/ml or >10% bands. INR, International Normalized Ratio; APTT, activated partial thromboplastin time. |
| Sepsis   - Suspected infection + >2 SIRS-criteria |
| Severe sepsis   - Sepsis *or* documented infection + either hypotension, hypoperfusion *or* organ dysfunction |
| Septic shock   - Persisting hypotension despite adequate volume resuscitation (500-1000 ml of crystalloid given within 30 minutes) plus either hypoperfusion or organ dysfunction |
| Criteria for hypotension, hypoperfusion and organ dysfunction |
| Hypotension   - Systolic blood pressure <90 mm Hg or mean arterial pressure <70 mm Hg |
| Hypoperfusion   - Blood lactate >3 mmol/l or + >1 mmol/l above the upper reference limit, *or*, base excess <-5 mmol/l |
| Organ dysfunction, either of   - Respiratory; PaO2/FiO2 <33 kPa (corresponding to 86% saturation on air breathing) *or* PaO2/FiO2 <27 kPa (corresponding to 78% saturation on air breathing) if the lung is the focus of infection. - Renal; <0.5 ml urine/kg/2 hours despite adequate volume resuscitation. - Hematologic; Thrombocytes <100 x 106/ml, or INR >1.5, or APTT >60 seconds. - Cerebral; Acute change of mental status. - Hepatic; Serum bilirubin >45 µmol/l. |

Severe sepsis or septic shock was diagnosed using the 2011 Swedish consensus definition and criteria with two exceptions: 1. Activated partial thromboplastin time, APTT, was not routinely analyzed. 2. In the triage system Rapid Emergency Triage and Treatment System (RETTS) used by the EMS and in our hospital, the Reaction Level Scale, RLS, was used for assessing mental status. We used RLS >2 as an equivalent to “acute change of mental status”.

For diagnosis of SIRS, values at baseline were used. Baseline SIRS was defined as the highest pulse rate, highest respiratory rate, highest or lowest leukocyte count and highest or lowest temperature documented by the EMS, in the ED before initiation of intravenous antibiotic therapy.
